# Combinatorial modulation of initial codons for improved zeaxanthin synthetic pathway efficiency in *Escherichia coli*

**DOI:** 10.1002/mbo3.930

**Published:** 2019-09-18

**Authors:** Zaiqiang Wu, Dongdong Zhao, Siwei Li, Junsong Wang, Changhao Bi, Xueli Zhang

**Affiliations:** ^1^ Center for Molecular Metabolism School of Environmental and Biological Engineering Nanjing University of Science and Technology Nanjing China; ^2^ Tianjin Institute of Industrial Biotechnology Chinese Academy of Sciences 32 West 7th Ave, Tianjin Airport Economic Park Tianjin 300308 China

**Keywords:** initiation codon, lycopene, metabolic pathway balance, zeaxanthin, β‐carotene

## Abstract

A balanced and optimized metabolic pathway is the basis for efficient production of a target metabolite. Traditional strategies mostly involve the manipulation of promoters or ribosome‐binding sites, which can encompass long sequences and can be complex to operate. In this work, we found that by changing only the three nucleotides of the initiation codons, expression libraries of reporter proteins RFP, GFP, and lacZ with a large dynamic range and evenly distributed expression levels could be established in *Escherichia coli* (*E. coli*). Thus, a novel strategy that uses combinatorial modulation of initial codons (CMIC) was developed for metabolic pathway optimization and applied to the three genes *crtZ, crtY,* and *crtI* of the zeaxanthin synthesis pathway in *E. coli*. The initial codons of these genes were changed to random nucleotides NNN, and the gene cassettes were assembled into vectors via an optimized strategy based on type II restriction enzymes. With minimal labor time, a combinatorial library was obtained containing strains with various zeaxanthin production levels, including a strain with a titer of 6.33 mg/L and specific production value of 1.24 mg/g DCW—a striking 10‐fold improvement over the starting strain. The results demonstrated that CMIC was a feasible technique for conveniently optimizing metabolic pathways. To our best knowledge, this is the first metabolic engineering strategy that relies on manipulating the initiation codons for pathway optimization in *E. coli*.

## Graphical abstract

In this work, we found that by changing only the initiation codons, expression libraries of reporter proteins RFP, GFP, and lacZ, with a large dynamic range and evenly distributed expression levels, could be established in *Escherichia coli*. Subsequently, a novel combinatorial modulation of initial codon (CMIC) strategy was developed for metabolic pathway optimization. CMIC was applied to the zeaxanthin synthesis pathway in *E. coli*, which achieved a nearly 10‐fold increase in zeaxanthin production.

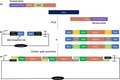

## 1 INTRODUCTION

Many natural products are structurally too complex to be economically synthesized through purely chemical means, while also being present in low quantities in their natural sources (Pitera, Paddon, Newman, & Keasling, 2007; Wu et al., 2016). With the development of metabolic engineering technologies and synthetic biology tools, microbial cell factories were constructed to heterologously produce such chemicals and natural products (Leonard, Lim, Saw, & Koffas, 2007; Pitera et al., 2007; Watts, Mijts, & Schmidt‐Dannert, 2005).

An efficient synthetic pathway for the target product is a *sine qua non* for the successful development of a cell factory. However, unwanted byproducts and intermediates can sometimes be accumulated in an unbalanced pathway and affect the pathway efficiency and final product yield (Berry, Dodge, Pepsin, & Weyler, 2002; Keasling, 2010; Xu, Gu, et al., 2013; Zhu, Lawman, & Cameron, 2002). Furthermore, some intermediates or heterologous enzymes are cytotoxic to the host cell once accumulated (Barbirato, Grivet, Soucaille, & Bories, 1996; Harcum & Bentley, 1999; Pitera et al., 2007). Therefore, balancing the metabolic pathway is a universal strategy for cell factory engineering (Pitera et al., 2007), which normally involves optimizing the transcriptional and translational levels of pathway genes (Brynildsen, Wong, & Liao, 2005; Jin et al., 2017; Xu, Gu, et al., 2013; Xu, Li, Zhang, Stephanopoulos, & Koffas, 2014; Xu, Vansiri, Bhan, & Koffas, 2012). To optimize a pathway with multiple enzymes, it is ideal to analyze all possible expression levels of pathway genes in a combinatorial fashion. Several strategies and methods can be used to construct plasmid libraries encoding as many of the possible expression level combinations as possible (Chen et al., 2005; Zaslaver et al., 2006), or directly modulate multiple genes on the chromosome (Zhu et al., 2017). Published approaches include the modulation of promoters (Cox, Surette, & Elowitz, 2007; Xu, Rizzoni, Sul, & Stephanopoulos, 2017) and ribosome‐binding sites (RBSs) (Salis, Mirsky, & Voigt, 2009), manipulation of intergenic regions (Pfleger, Pitera, Smolke, & Keasling, 2006), dynamic promoter regulation (Farmer & Liao, 2000; Xu, Bhan, & Koffas, 2013; Zhang, Carothers, & Keasling, 2012), organelle compartmentalization of pathways (Avalos, Fink, & Stephanopoulos, 2013; Farhi et al., 2011), and modulation of DNA copy numbers (Juminaga et al., 2012). These strategies involved direct employment of regulators and could generate a wide dynamic range to benefit for pathway optimization, but they also require manipulation of relatively long sequences and can be complex to operate, which makes more convenient strategies highly desirable.

The initiation codon contains only three nucleotides, yet it significantly affects the gene expression strength at the translational level (Looman et al., 1987). ATG is the most common codon, but GTG, and more rarely TTG, is also employed by some genes (Aiba et al., 1984; Danchin, Guiso, Roy, & Ullmann, 1984). It was found that GTG has a lower translation initiation efficiency than ATG, and sometimes, ATG was used to replace GTG to increase target gene expression (Reddy, Peterkofsky, & McKenney, 1985), which suggested that various codons among the exhaustive 64 combinations in an NNN library might lead to different initiation efficiency. Thus, it might be feasible to gradually modulate gene expression by changing the initiation codons. In this work, we found that the expression of reporter proteins RFP, GFP, and lacZ could be modulated by changing only the three nucleotides of their initiation codons. As intended, the expression libraries with genes initiated by random NNN codons indeed showed a large dynamic range and mostly evenly distributed expression levels. Due to the simplicity of manipulating only three or fewer nucleotides of the initiation codon, future methods using our approach might be much simpler than current strategies. Thus, a novel strategy of combinatorial modulation of initial codons (CMIC) was developed for metabolic pathway optimization in this work, which offers great flexibility at minimal costs of experimental materials and time.

Carotenoids were reported to be beneficial for the treatment and prevention of many diseases (Bourcier de Carbon, Thurotte, Wilson, Perreau, & Kirilovsky, 2015; Farmer & Liao, 2000; Sajilata, Singhal, & Kamat, 2008), acting as effective antioxidants (Sies & Stahl, 1998), as well as inhibitors of age‐related macular degeneration (Moeller, Jacques, & Blumberg, 2000; Nishino, Murakoshi, Tokuda, & Satomi, 2009) and cataract formation (Moeller et al., 2000). Zeaxanthin, which is derived from the central carotenoid synthesis intermediates lycopene and β‐carotene, was reported to be vital in protecting the retina from damage (Stahl & Sies, 2005; Thomson et al., 2002) and also is regarded as an antioxidant (Krinsky & Johnson, 2005; Whitehead, Mares, & Danis, 2006). It is synthesized from phytoene via a short pathway comprising the enzymes crtI (phytoene desaturase), crtY (lycopene β‐cyclase), and crtZ (β‐carotenoid hydroxylase) (Sun et al., 2014; Zhao et al., 2013). In this work, the zeaxanthin synthesis pathway containing three gene products was optimized using CMIC to illustrate the application of this novel technique in *E. coli*.

## 2 MATERIALS AND METHODS

### 2.1 Strains, media, and culture conditions

The strains and plasmids used in this study are listed in Table A1. *E. coli* was cultured at 37°C in Lysogeny broth (10 g/L Difco tryptone, 5 g/L Difco yeast extract, and 10 g/L NaCl). The carotenoid fermentation medium was composed of (per liter) 10 g tryptone, 5 g yeast extract, and 10 g NaCl; 2% glycerol (v/v) was added to LB (Lysogeny broth) + glycerol. Apramycin sulfate (50 mg/L; Ruitaibio), chloramphenicol (34 mg/L; Solarbio), ampicillin (100 mg/L; Solarbio), kanamycin (50 mg/L; Solarbio), or β‐D‐1‐thiogalactopyranoside (IPTG, 1 mmol/L; Solarbio) were added to the media, where appropriate. Plasmids were extracted using the Bacterial Genomic DNA Miniprep Kit (Axygen Biosciences). Polymerase chain reaction (PCR) products were digested with DpnI for 0.5 hr at 37°C and then purified using a SanPrep Gel Extraction Kit (Sangon Biotech). Plasmids and PCR products were sequenced using Sanger sequencing (GenScript Co., Ltd).

### 2.2 Construction of the reporter expression libraries pNNNrfp, pNNNgfp, and pNNNlacZ

The primers pBBR1‐rfp‐F and pBBR1‐rfp‐R were used to amplify the backbone of pNNNrfp from plasmid pBBR1‐rfp, and the *rfp* gene was cloned into the pNNNrfp plasmid with kanamycin‐resistance cassette and pBBR1 replication origin, driven by the constitutive promoter BBa J23100 (Table A2). The initiation codon library NNN was embedded into the forward primer pBBR1‐rfp‐F. The resulting PCR product was digested with *Dpn*I to eliminate the PCR template and self‐ligated using Golden Gate DNA assembly (Hillson, Rosengarten, & Keasling, 2012).

The GFP expression library pNNNgfp contained quite different components from those used to construct pNNNrfp, to construct pNNNgfp, the backbone fragment containing a pMB1 origin of replication and an apramycin‐resistance cassette was amplified from plasmid p034apr using the primer pair pMB1_apr_F and pMB1_apr_R; the constitutive promoter P46 (Table A2) was amplified from the strain M1‐46 using the primers p46‐up and GFP_RBS‐down containing the randomized initiation codon NNN; the *gfp* gene was cloned from plasmid pQE60‐gfp.

To construct the *lacZ* library pNNNlacZ, the backbone fragment comprising a pMB1 origin of replication and an apramycin‐resistance cassette was amplified from plasmid p034apr using the primer pair pMB1_apr_F and pMB1_apr_R; the constitutive P46 promoter (Table A2) was amplified from strain M1‐46 using the primers p46‐up and lacZ_RBS‐down containing the randomized initiation codon NNN; the *lacZ* gene was cloned from *E. coli* MG1655 using the primers LacZ_F and LacZ_R. The resulting plasmid libraries pNNNrfp, pNNNgfp, and pNNNlacZ were transferred into *E. coli* DH5α (CWBIO) and selected overnight on the LB plates with the corresponding antibiotics. The resulting colonies were used for expression analysis. All primers used in library construction are listed in Table A3 and the sequencing primers in Table A4.

### 2.3 Construction of pCrtZYIlib libraries for combinatorial modulation of initial codons

To construct the combinatorial modulated plasmid library, primers crt‐F and crt‐R were used to amplify the backbone of the pCrtZYIlib from the plasmid pYL‐crtZYI with a pSC101 replication origin and a chloramphenicol‐resistance cassette; promoter 36 was amplified from the strain M1‐36 using primers P36‐F and P36‐R; and the *crtZ* gene was amplified from the plasmid pYL‐crtZYI using primers crtZ‐F and crtZ‐R, with the randomized initiation codon NNN embedded in the primer crtZ‐F. The *crtY* gene was amplified from the plasmid pYL‐crtZYI using the NNN‐containing primers crtY‐F and crtY‐R. The *crtI* gene was amplified from the plasmid pYL‐crtZYI using primers crtI‐F and crtI‐R with the same strategy. All the DNA fragments were digested using *Dpn*I at 37°C for 0.5 hr and ligated using the Golden Gate method (Hillson et al., 2012). All primers used in library construction are listed in Table A3 and the sequencing primers in Table A4.

### 2.4 Zeaxanthin production levels of different clones from the CMIC library

All CMIC library colonies were scraped from the plates and pooled for plasmid DNA extraction. The resulting plasmid library was transferred into the chassis strain PHY01 and grown overnight on LB/chloramphenicol plates. The resulting single colonies were picked from the plates and used to inoculate 15 mm × 100 mm tubes containing 3 ml of LB with 34 mg/L chloramphenicol and grown at 37°C and 250 rpm overnight. Aliquots comprising 100 μl of the resulting seed cultures were used to inoculate 100‐ml flasks containing 10 ml LB + 2% (v/v) glycerol carotenoid fermentation medium, and grown aerobically at 30°C and 250 rpm for 48 hr. The resulting fermentation cultures were collected for measurement of carotenoid production and biomass (OD_600 nm_).

### 2.5 RFP and GFP fluorescence measurement

The RFP‐ and GFP‐expressing colonies were picked and transferred into 15 mm × 100 mm tubes containing 3 ml LB with 50 mg/L kanamycin and 50 mg/L apramycin, respectively, and grown at 37°C and 250 rpm overnight. The cultures were then inoculated into 15 mm × 100 mm tubes containing 3 ml LB with 50 mg/L with the same antibiotics and grown at 37°C and 250 rpm for 20 hr. Subsequently, 50 μl samples of each culture were transferred into individual wells of a 96‐well plate and diluted four times with LB. The blank control was 200 µl of pure LB. The optical density at 600 nm (OD_600 nm_) was measured for determining the biomass concentration using an SP‐723 spectrophotometer (Spectrum SHANGHAI). Fluorescence was measured at a gain of 60, using an excitation wavelength of 585 nm emission wavelength of 620 nm for RFP, 488 and 520 nm, respectively, for GFP, using an Infinite M200 Pro ELISA spectrometer (Tecan).

### 2.6 Measurement of* lacZ* expression

A quantitative estimate of lacZ expression was obtained by measuring the β‐galactosidase activity using ortho‐nitrophenyl‐β‐D‐galactopyranoside (ONPG; Sigma) as a colorimetric substrate. Colonies grown on LB/apramycin plates at 37°C overnight were used to inoculate 15 mm × 100 mm tubes containing 4 ml LB with 50 mg/L apramycin and cultured for 4 hr at 37°C. The resulting actively growing mid‐log cultures were incubated on ice for 20 min to stop the growth and collected by centrifugation at 1,500 *g* and 4°C for 10 min. The resulting cell pellet was resuspended in the same volume of Z buffer (per 50 ml: 0.80 g Na_2_HPO_4_∙7H_2_O [0.06 M], 0.28 g NaH_2_PO_4_∙H_2_O [0.04 M], 0.5 ml 1 M KCl [0.01 M], 0.05 ml 1 M MgSO_4_ [0.001 M], 0.135 ml β‐mercaptoethanol (BME) [0.05 M], pH 7.0), and the cell density was measured at OD_600 nm_ using Z buffer as the blank. For enzyme activity measurements, 50 μl of cell suspension was added to 950 μl of Z buffer, permeated by adding 100 μl chloroform and 50 μl 0.1% SDS, and whirled for 30 S with a vortex mixer. The reaction was started by adding 200 μl 4 mg/ml ONPG and vortexing, continued for 1.5 min at 28°C, and stopped by adding 0.5 ml 1 M Na_2_CO_3_ solution. The absorbance at 420 and 550 nm was measured for each sample. The units of activity were calculated using the formula Miller Units = 1,000 × [(OD_420_ – 1.75 × OD_550_)]/(*T* × *V* × OD_600_), where *T* = reaction time in minutes and *V* = volume of culture used in the assay in ml (Kumari, Panesar, & Panesar, 2004; Otsuka, Nakabeppu, & Sekiguchi, 1985).

### 2.7 Measurement of carotenoid production of clones from the CMIC library

An aliquot comprising 1 ml of each culture was harvested by centrifugation at 12,000 *g* for 5 min, suspended in 1 ml acetone, incubated at 55°C for 15 min in dark, and centrifuged at 12,000 *g* for 10 min. The acetone supernatants containing the carotenoids were transferred into fresh tubes for HPLC analysis. The HPLC was conducted on a Technologies Series 1200 system (Agilent) equipped with a VWD detector at 476 nm and a Symmetry C18 column (250 mm × 4.6 mm, 5 μm, Waters). A mixed gradient flow elution at a flow rate of 0.8 ml/min at 30°C containing mobile phase C (methanol, acetonitrile, and dichloromethane at 21:21:8, by volume) and phase D (10% methanol [v/v]) was employed to separate the analytes as described previously (Li et al., 2017). The dry cell weight (DCW) was calculated from the optical density at 600 nm using the empirical formula 1 OD_600_ = 0.323 g DCW/L. The results are shown as the means ± *SD* of three repeated experiments.

### 2.8 Total RNA extraction and qRT‐PCR analysis

In order to investigate the relationship between non‐ATG initial codons and the transcriptional expression levels of the key carotenoid synthetic pathway genes, two representative strains PHY01(pCrtZYI7) and PHY01(pCrtZYI9) and the control strain PHY01(pCrtZYIATG) were chosen to analyze the strength of the gene expression through real‐time qPCR (RT‐qPCR). Total RNA was extracted and prepared using the RNAprep Pure Plant Kit (Qiagen, DP441). For preparing the cDNA, reverse transcription was conducted using the *TransScript* II All‐in‐One First‐Strand cDNA Synthesis SuperMix for qPCR (One‐Step gDNA Removal) Kit (TransGen Biotech, AH341), which included the procedure of one‐step genomic DNA (gDNA) removal. The qPCR was analyzed using the *TransStart* Top Green qPCR SuperMix Kit (TransGen Biotech, AQ131) on the Bio‐Rad CFX Connect^TM^ Real‐Time PCR System (Bio‐Rad, CFX96 Touch). The primers that are used for the RT‐qPCR analysis are shown in Table A5, and 16S rRNA gene was used as the endogenous reference gene. The relative gene transcript level was calculated using the comparative critical threshold cycle method ($2^{-\Delta \Delta C_{t}}$). The data were presented as mean ± *SD* (standard deviation) of triplicate experiments.

### 2.9 Protein extraction and sample preparation

To collect total proteins for mass spectrometry analysis, the cell protein extraction procedure was as follows: (a) Prepare 150‐ml fermentation medium of the *E. coli* PHY01(pCrtZYIATG), PHY01(pCrtZYI7), and PHY01(pCrtZYI9), and then the cells are harvested by centrifugation at 3,500 *g* for 10 min; (b) dissolve the cell pellet using 15 ml PBS buffer (pH 7.2) and repeat this step three times; (c) discard the supernatant and collect the pellet for the next step; (d) the collected pellet is dissolved using the 10 ml protein lysate (8 M urea, 1% DTT) and mixed well; (e) the suspension is crushed with the ultrasonic breaker (Scientz‐IID) for 10 min under ice‐bath condition; (f) the crushed suspension is centrifuged at 8,000 *g* for 15 min at 18°C; and (g) collect the supernatant into the 2‐ml centrifugal tube and repeat this step once, and the samples are stored at −80°C for analysis or protein mass spectrometry.

### 2.10 Statistical analysis and analytical techniques

The significance of differences between mean values of control and test samples was compared using Student's *t* test in the open‐source software suite “R” (http://cran.r-project.org/). Differences with *p* < .05 were regarded as obvious, *p* < .01 as significant, and *p* < .001 as very significant. The SDS‐PAGE was run using the commercially purchased SurePageTM Gels (GenScript). The protein mass spectrometry was performed using the Orbitrap Fusion Lumos Tribrid Mass Spectrometer (LC‐MS) (Thermo Fisher), and the methods could be referred to references (Espadas, Borras, Chiva, & Sabido, 2017; Li, Zhou, Xiao, Li, & Tian, 2018).

## 3 RESULTS AND DISCUSSION

### 3.1 The expression intensity of reporter protein expression libraries with randomized NNN initiation codons

**Figure 1:**
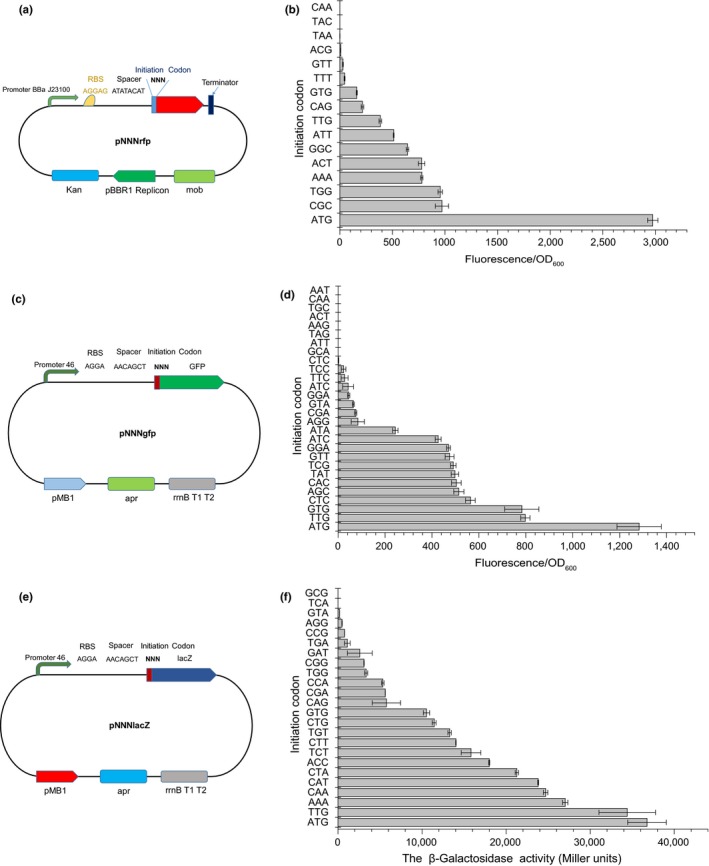
A schematic diagram of the construction of the RFP, GFP, and lacZ plasmid libraries and the corresponding experimental results. (a) The schematic diagram of pNNNrfp construction; (b) the fluorescence intensity (fluorescence/OD_600 nm_) of selected pNNNrfp strains; (c) the schematic diagram of pNNNgfp construction; (d) the fluorescence intensity (fluorescence/OD_600 nm_) of selected pNNNgfp strains; (e) the schematic diagram of pNNNlacZ construction; and (f) the β‐galactosidase activity (Miller Units) of selected pNNNlacZ strains. The data represent the means of three experiments, and the error bars represent their standard deviations.

To determine whether the expression of genes could be gradually modulated by changing their initiation codons and study the relationship between expression levels and initiation codons, reporter libraries individually expressing RFP, GFP, and lacZ with randomized NNN initiation codons were constructed in *E. coli*. The RBS core region of the pNNNrfp was AGGAG and the spacer sequence between the RBS and the initiation codon was ATATACAT (Figure 1a), which was reported to be essential for translation initiation (Chen, Bjerknes, Kumar, & Jay, 1994). Colonies with visually apparent diversity of expression levels were selected semi‐randomly from the pNNNrfp library on LB plates and subjected to growth and fluorescence measurement. The RFP expression levels were determined by calculating the specific fluorescence per OD_600 nm_.

**Figure A1:**
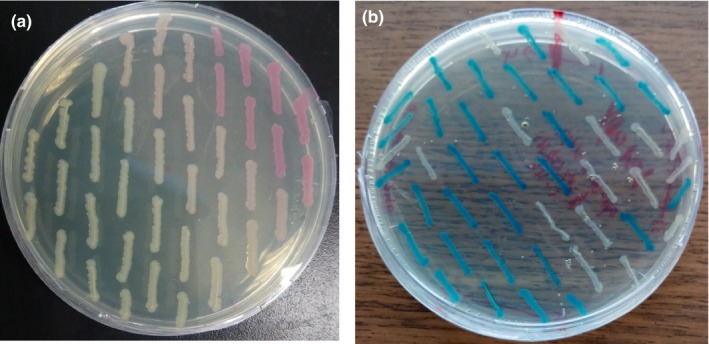
The pictures of pNNNrfp library and pNNNlacZ library on LB plates. (a) pNNNrfp library on LB plates and (b) pNNNlacZ library on LB plates

The specific fluorescence of selected strains from the pNNNrfp library is shown in Figure 1b. While ATG still gave the strongest expression, the canonical initial codons of GTG and TTG had an expression strength 5% and 13% of that of ATG, respectively, which was comparable to previous reports (Beard & Spindler, 1996; Rhee, Yang, Lee, & Park, 2004; Stenström, Holmgren, & Isaksson, 2001; Tang et al., 2017). It was interesting that some of the non‐natural codons had relatively high expression levels, whereby CGC, TGG, AAA, and ACT had 26%–33% of the efficiency of ATG; GGC, ATT, and CAG initiated translation with an efficiency of 7.2%–21.6%; TTT, GTT, ACG, and TAA showed 0.1%–1.5% relative efficiency, while TAC and CAA had nondetectable fluorescence intensity. These results suggested that the randomized NNN initiation codon library had mostly evenly distributed expression levels. Moreover, even not counting the strains with nondetectable fluorescence, the library had a large dynamic range of around 3,000‐fold. A photograph of the pNNNrfp library colonies on an LB plate is shown in Figure A1a. The library of the initiation codon library of *rfp* gene (pNNNrfp) that we obtained was around four thousand colonies, and the coverage of the initiation codon library of *rfp* gene (pNNNrfp) was around 62‐fold.

To better study the quality of the expression libraries with randomized initiation codons, and investigate whether there is a universality of the relationship between expression levels and initiation codons in different contexts, reporter libraries with GFP and lacZ were also constructed and analyzed. Different RBSs, spacers, resistance markers, and constitutive promoter were used for investigating the initiation codons in different genetic contexts. In addition, we have checked the sequences of the genes we have used in this research to see whether there was in‐frame ATG, GTG, or TTG codons within the UTR region of the three reporter genes and the *crt* genes. As a result, none of ATG, GTG, and TTG codons were found. Additionally, there are no internal nature initiation codons that could shift the initial codons. The RBS core region of pNNNgfp was AGGA, and spacer sequence was AACAGCT (Figure 1c). The library of the combinatorial initiation codon library of *gfp* gene (pNNNgfp) was around 5,500 colonies, and the coverage of the initiation codon library of *gfp* gene (pNNNgfp) was nearby 86‐fold. While both natural initiation codons GTG and TTG were present from the pNNNgfp library, which had an expression strength 61% and 62% of that of ATG, respectively (Figure 1d), translation levels initiated by the non‐natural start codons CTC, AGC, CAC, TAT, TCG, GTT, GGA, ATC, and ATA were high and evenly distributed, ranging from 19.0% to 43.9% of that of ATG. The remaining codons AGG, CGA, GTA, GGA, ATC, TTC, TCC, and CTC had translation efficiencies in the range of 0.01%–6.5% of that of ATG, which was similar to previous reports (Hecht et al., 2017; O'Connor, Gregory, Rajbhandary, & Dahlberg, 2001; Sussman, Simons, & Simons, 1996). No GFP fluorescence was detected with the start codons GCA, ATT, TAG, AAG, ACT, TGC, CAA, and AAT (Figure 1d).

The RBS core region of pNNNlacZ was AGGA, and the spacer sequence was AACAGCT (Figure 1e). The library of the combinatorial initiation codon library of *lacZ* gene (pNNNlacZ) was around six thousand colonies, and the coverage of the initiation codon library of *lacZ* gene (pNNNlacZ) was nearby 94‐fold. The β‐galactosidase activity (Miller Units) of individual colonies was measured to evaluate *lacZ* gene expression. Among the three reporter libraries, the pNNNlacZ library had the most evenly distributed expression levels, and the natural codons GTG and TTG were also present, which had an expression strength 28.6% and 93.6% of that of ATG, respectively (Figure 1f). The artificial start codons AAA, CAA, CAT, CTA, ACC, TCT, CTT, TGT, and CTG had 31.1%–73.5% of the strength of ATG, while those of the artificial start codons CAG, CGA, CCA, TGG, CGG, GAT, TGA, CCG, AGG, and GTA ranged from 0.5% to 15.7%. Interestingly, the stop codon, TGA, was found to have translation initiation efficiency of 3.0% of that of ATG (Figure 1f). Only TCA and GCG had no detectable increase in β‐galactosidase activity over the control. Colonies of the pNNNlacZ library on an LB plate are shown in Figure A1b.

**Figure A2:**
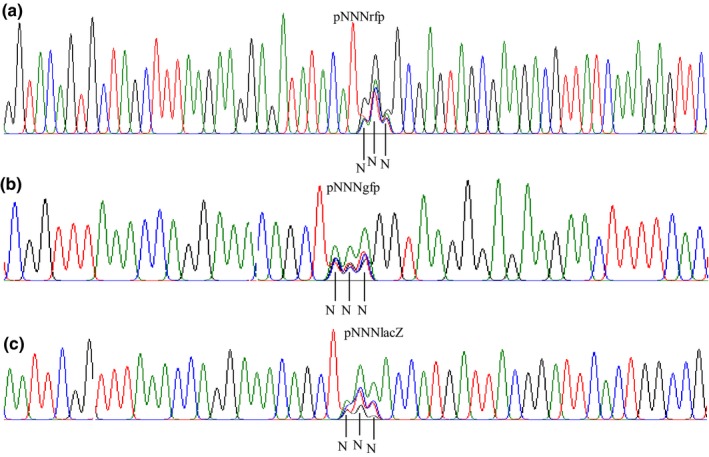
The frequency of each non‐natural start codon in the reporter expression libraries. (a) The frequency of each non‐natural start codon in the reporter expression libraries of pNNNrfp; (b) the frequency of each non‐natural start codon in the reporter expression libraries of pNNNgfp; and (c) the frequency of each non‐natural start codon in the reporter expression libraries of pNNNlacZ. The green peak represents the base of A, the blue peak represents the base of C, the red peak represents the base of T, and the black peak represents the base of G

For studying the frequency of each non‐natural start codon in the reporter expression libraries of pNNNrfp, pNNNgfp, and pNNNlacZ, the plasmid libraries were sequenced with normal Sanger sequencing method. And the resulted ab format reporting files contain the chromatograph, the area of the peaks for A, T, C, or G semi‐quantitatively represents the frequency of bases called for a position. The regions containing initial codons are adapted into Figure A2 (a, b, and c). The green peak represents the base of A, the blue peak represents the base of C, the red peak represents the base of T, and the black peak represents the base of G. These results indicated that all four bases were almost evenly represented in the initial coding region, which suggested a good coverage of the initial codon libraries.

The experimental results of the reporter protein (RFP, GFP, lacZ) expression libraries with the randomized NNN initiation codons indicated that the noncanonical start codons did not produce the same relative expression levels in different contexts. It seems that the translational initiation efficiency of initial codons has a very vague conservation. Therefore, the reporter expression strength had little predictive value. Although we did not find all the 64 possible codon triplets in each reporter library, the results indicated that some of the non‐natural initiation codons could initiate translation relatively efficiently. Thus, the expression of gene could be modulated in a gradual fashion by changing only their initiation codons, and high‐quality expression libraries could be established by replacing ATG with the NNN nucleotide oligo.

### 3.2 Development of a combinatorial modulation of initial codons technique for metabolic pathway optimization

**Figure 2:**
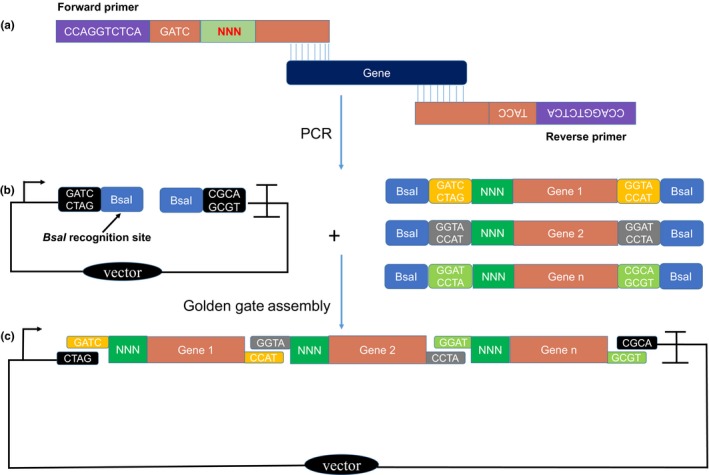
The construction process of a CMIC library. (a) Primer design for modulating gene expression. The primers contained a *Bsa*I recognition site, optimized linkers, and the random initiation codon NNN; and (b) assembly of DNA cassettes containing modulated genes. A ready‐made vector part was used to assemble the cassettes; and (c) the structure of the CMIC library. This library contained all genes with randomized initiation codons NNN in various combinations

**Figure A3:**
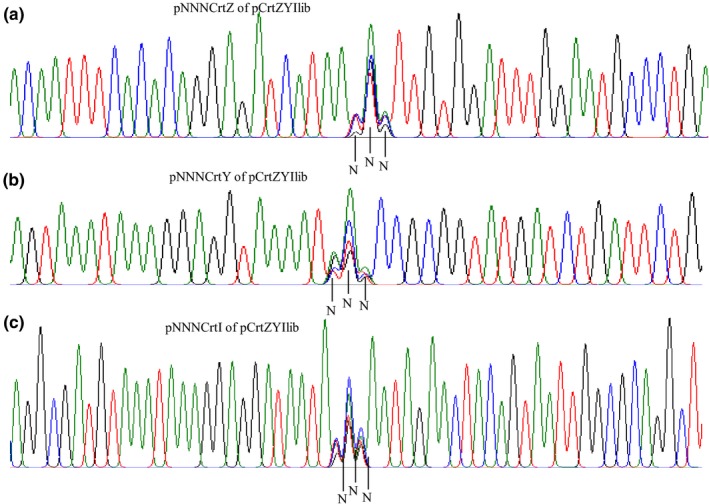
The frequency of each non‐natural start codon in the expression libraries of pCrtZYIlib. (a) The frequency of each non‐natural start codon in the expression libraries of pNNNCrtZ; (b) the frequency of each non‐natural start codon in the expression libraries of pNNNCrtY; and (c) the frequency of each non‐natural start codon in the expression libraries of pNNNCrtI. The green peak represents the base of A, the blue peak represents the base of C, the red peak represents the base of T, and the black peak represents the base of G

Since random initiation codons could be employed to generate gene expression libraries, we used the CMIC technique as a simple and feasible method to modulate and optimize the expression of multiple genes simultaneously (Figure 2). Variably regulated genes were obtained by PCR amplification with extended primers, in which the initiation codon nucleotides NNN were embedded at the 5′ ends. Specifically designed linkers for type II restriction enzymes were also embedded into the primers to ensure the assembly pattern and efficiency. Using the Golden Gate assembly method (Hillson et al., 2012), DNA cassettes containing the pathway genes were assembled into the vector backbone to form an expression plasmid. With the above‐mentioned method, the frequency of the four bases in the initial codons of pCrtZYIlib libraries was obtained by sequencing the mixture of the library with normal Sanger sequencing method. As illustrated in Figure A3 (a, b, and c), all four bases were almost evenly represented in the initial coding region, which suggested a good coverage of the initial codon libraries.

Since each gene had a random initiation codon, a combinatorial plasmid library with variably regulated pathway genes was created, which was subsequently introduced into dedicated hosts to be screened and selected for strains carrying optimized pathways. The vector backbone was universal for all reactions, providing a stable plasmid backbone. By incorporating fixed linkers and regulatory elements into the primers for gene amplification, this method varies only the actual PCR primer sequences of pathway genes (Figure 2).

### 3.3 Application of the CMIC technique to improve the efficiency of the zeaxanthin synthesis pathway

The experimental results of the reporter protein (RFP, GFP, lacZ) expression libraries with the randomized NNN initiation codons indicated that the noncanonical start codons did not produce the same relative expression levels with the three reporter genes in *E. coli*. Therefore, the reporter expression strength had no predictive value for the expression of the *crtZ*, *crtY,* and *crtI* genes in the zeaxanthin pathway. Consequently, we adopted a strategy of creating a de novo codon library for each *crt* gene in *E. coli*.

**Figure 3:**
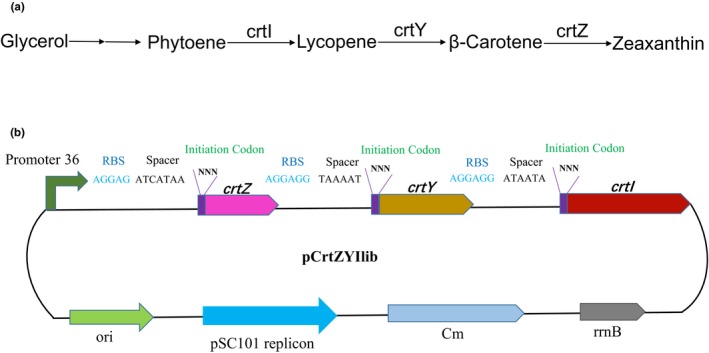
The structure of the crtZYI CMIC library pCrtZYIlib. (a) Schematic diagram of the zeaxanthin biosynthesis pathway; and (b) the structure of the crtZYI CMIC library pCrtZYIlib

**Figure A4:**
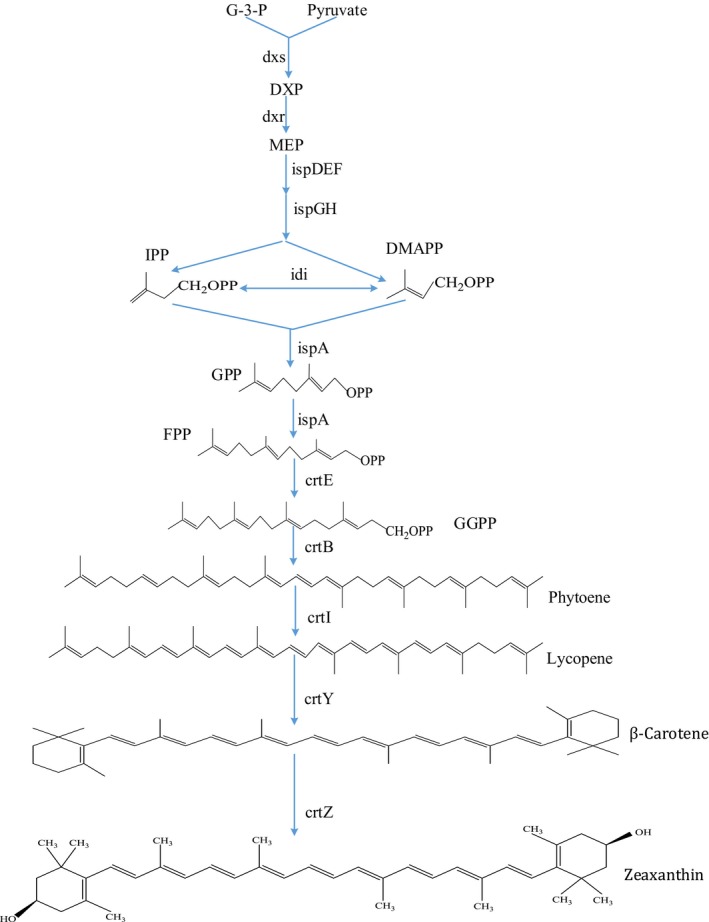
Biosynthesis pathway of zeaxanthin of the engineering strain PHY01(pCrtZYIlib). G‐3‐P, glyceraldehyde; DXP, 1‐deoxy‐D‐xylulose 5‐phosphate; MEP, methyl‐d‐erythritol 4‐phosphate; IPP, isopentenyl pyrophosphate; DMAPP, dimethylallyl pyrophosphate; GPP, geranyl pyrophosphate; FPP, farnesyl pyrophosphate; GGPP, geranylgeranyl pyrophosphate

Zeaxanthin is synthesized from phytoene via a short pathway comprising the enzymes crtI (phytoene desaturase), crtY (lycopene β‐cyclase), and crtZ (β‐carotenoid hydroxylase) (Figures 3a and A4). This synthesis pathway containing three gene products was optimized using CMIC to demonstrate a practical application of this novel technique (Figure 3b). The chassis strain PHY01 (Table A1) producing the precursor of the zeaxanthin synthesis pathway, phytoene, was constructed previously using classic metabolic engineering strategies (Lu et al., 2012; Sun et al., 2014; Zhao et al., 2013). Using the CMIC strategy, primers were designed to amplify *crtZ*, *crtY,* and *crtI* from the plasmid pYL‐crtZYI (Table A1). The designed sequences contained *Bsa*I recognition sites (GGTCTC) and specific 4‐bp linkers, as well as the random nucleotides NNN at the 5′ end of the forward primer to replace the original initiation codons. In the Golden Gate assembly reaction, the ready‐made plasmid backbone was mixed with the CDS parts *crtZ*, *crtY,* and *crtI*. After the Golden Gate assembly reaction, the plasmid library pCrtZYIlib was produced, which contained the *crtZ*, *crtY,* and *crtI* coding sequences with different initial codons in various combinations (Figure 3b).

**Figure 4:**
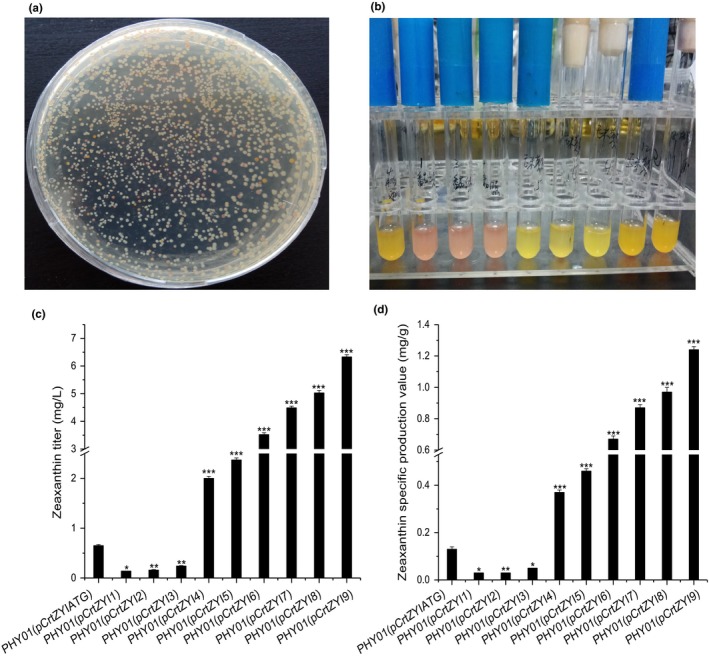
The photographs, zeaxanthin titers, and specific production values of selected strains from the pCrtZYIlib random‐starting‐codon library. (a) Photographs of pCrtZYIlib library colonies on an LB plate; (b) selected strains after incubation in test tubes; (c) the zeaxanthin titer of control strain PHY01(pCrtZYIATG) and nine strains, designated PHY01(pCrtZYI1) to PHY01(pCrtZY9), in fermentation medium (LB + 2% glycerol [v/v]); and (d) the specific zeaxanthin production value of the control strain PHY01(pCrtZYIATG) and the nine strains PHY01(pCrtZYI1) to PHY01(pCrtZY9) in fermentation medium (LB + 2% glycerol [v/v]). The data represent the means of three experiments, and the error bars represent their standard deviations. The significance of differences is determined using Student's t‐test and presented as p‐values. The asterisks indicate a significant difference compared with the control (***p < .001; **p < .01; *p < .05)

The pCrtZYIlib plasmid library of around thirty thousand strains was obtained in *E. coli* DH5α on solid LB plates with 34 mg/L chloramphenicol and was subsequently transferred into the chassis strain PHY01 to obtain a combinatorial zeaxanthin production library of around thirty thousand colonies. The precursors and intermediates of the zeaxanthin pathway have different colors, with the colorless phytoene, red lycopene, orange β‐carotene, and golden yellow zeaxanthin (Figure 4a). Thus, high zeaxanthin‐producing strains could be crudely prescreened visually based on the color of the colonies (Figure 4b). After the first round of visual screen, the chosen strains were grown in 50‐ml flasks with 10 ml LB + 2% glycerol (v/v) at 30°C and 250 rpm for 48 hr before production analysis via HPLC.

Nine strains with representative zeaxanthin production levels designated as PHY01(pCrtZYI1) to PHY01(pCrtZYI9) were subjected for sequencing to determine the initiation codons of the *crtZ*, *crtY,* and *crtI* genes (Tables 1 and A6)*.* While PHY01(pCrtZYI1) to PHY01(pCrtZYI3) had lower specific zeaxanthin production values than the control strain containing the pCrtZYIATG plasmid with the original *crt* genes, PHY01(pCrtZYI4) to PHY01(pCrtZYI9) had 2.8‐ to 9.5‐fold increased zeaxanthin production (Table 1). The best strain PHY01(pCrtZYI9) produced 6.33 mg/L zeaxanthin with a specific production value of 1.24 mg/g DCW (Figure 4c,d), representing a 9.7‐fold and 9.5‐fold increase over the control strain (*p* < .001).

**Table 1:** Carotenoid production of selected strains from PHY01(pCrtZYIlib) with their corresponding initial codons of *crtZ, crtY,* and *crtI*

Strains^a^	Carotenoid production with different initiation codons by the multigenes of *crtZYI*								
	Zeaxanthin		*crtZ*	β‐Carotene		*crtY*	Lycopene		*crtI*
	titer^b^	spv^b^		titer	spv		titer	spv	
PHY01(pCrtZYIATG)	0.65 ± 0.02	0.13 ± 0.01	ATG	16.49 ± 0.09	3.35 ± 0.02	ATG	8.46 ± 0.06	1.72 ± 0.02	ATG
PHY01(pCrtZYI1)	0.14 ± 0.00	0.03 ± 0.00	TAG	0.00 ± 0.00	0.00 ± 0.00	AAC	17.20 ± 0.09	3.97 ± 0.05	ATG
PHY01(pCrtZYI2)	0.16 ± 0.01	0.03 ± 0.00	AAG	0.00 ± 0.00	0.00 ± 0.00	GAA	24.83 ± 0.14	4.72 ± 0.06	AAA
PHY01(pCrtZYI3)	0.24 ± 0.01	0.05 ± 0.00	CCA	0.00 ± 0.00	0.00 ± 0.00	CCT	24.14 ± 0.12	4.63 ± 0.05	ATG
PHY01(pCrtZYI4)	2.00 ± 0.04	0.37 ± 0.01	GGG	9.60 ± 0.04	1.76 ± 0.03	ACG	0.00 ± 0.00	0.00 ± 0.00	ATA
PHY01(pCrtZYI5)	2.37 ± 0.04	0.46 ± 0.01	AGC	5.62 ± 0.04	1.10 ± 0.01	ATG	6.19 ± 0.05	1.21 ± 0.02	GTA
PHY01(pCrtZYI6)	3.52 ± 0.07	0.67 ± 0.02	GTT	16.09 ± 0.10	3.07 ± 0.04	ATT	0.00 ± 0.00	0.00 ± 0.00	CTG
PHY01(pCrtZYI7)	4.49 ± 0.06	0.87 ± 0.02	TCA	11.93 ± 0.05	2.32 ± 0.02	GTG	0.00 ± 0.00	0.00 ± 0.00	TTG
PHY01(pCrtZYI8)	5.03 ± 0.09	0.97 ± 0.03	GAC	11.42 ± 0.06	2.21 ± 0.02	GTG	0.95 ± 0.01	0.18 ± 0.00	ACG
PHY01(pCrtZYI9)	6.33 ± 0.08	1.24 ± 0.02	ACG	16.06 ± 0.06	3.15 ± 0.04	ATT	0.00 ± 0.00	0.00 ± 0.00	CTG

^a^ Three repeated experiments were performed for every strain, and the error bars represented standard deviation.

^b^ Titer = mg/L, spv = specific production value = mg/g DCW.

It was perhaps surprising that none of the *crt* genes in the best strain PHY01(pCrtZYI9) had natural codons, indicating that the artificial codons regulated the zeaxanthin pathway more efficiently and with better balance than the original all‐ATG initiated pathway. The CMIC technique was therefore demonstrated to offer a feasible strategy for convenient metabolic pathway optimization.

An analysis of the concentrations of synthetic intermediates revealed that the low zeaxanthin‐producing strains PHY01(pCrtZYI1), PHY01(pCrtZYI2), and PHY01(pCrtZYI3) had high lycopene accumulation and no β‐carotene, suggesting that these strains had very unbalanced pathways so that the carbon flux was stopped at the first synthesis step. Conversely, most strains with improved zeaxanthin production had very low or no lycopene accumulation, but all accumulated some β‐carotene, indicating that it was beneficial to move the carbon flux to the second step of the synthesis pathway, which provided the direct substrate for zeaxanthin production.

### 3.4 CMIC technique modulated zeaxanthin synthesis pathway genes in translational level but not transcriptional level

To determine whether the non‐ATG initial codons influenced in the transcription level or translation level of these key genes, three experiments were performed, including real‐time qPCR (RT‐qPCR) analysis, SDS‐PAGE of total proteins, and protein mass spectrometry of total proteins.

**Figure A5:**
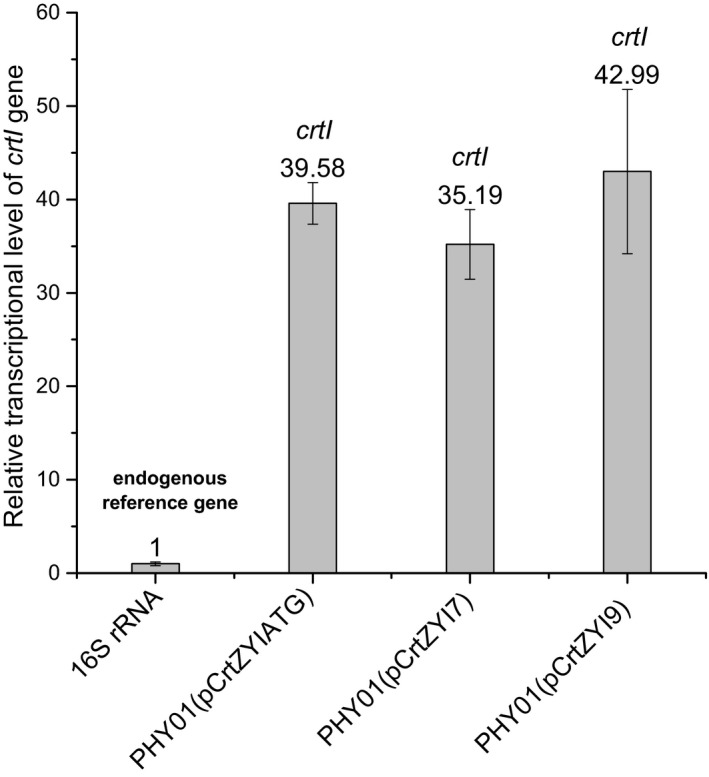
The relative transcriptional level of *crtI* gene in the strains of control strain PHY01(pCrtZYIATG), and experimental strains of PHY01(pCrtZYI7) and PHY01(pCrtZYI9). 16S rRNA was used as the endogenous reference gene. The data were shown as mean ± standard deviation (*SD*) of three independent experiments

**Figure A6:**
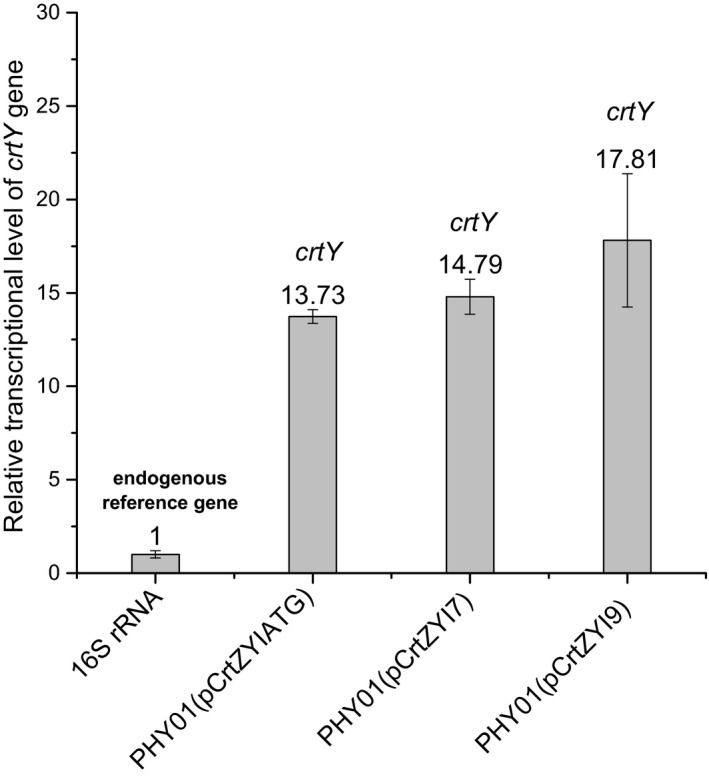
The relative transcriptional level of *crtY* gene in the strains of control strain PHY01(pCrtZYIATG), and experimental strains of PHY01(pCrtZYI7) and PHY01(pCrtZYI9). 16S rRNA was used as the endogenous reference gene. The data were shown as mean ± standard deviation (*SD*) of three independent experiments

**Figure A7:**
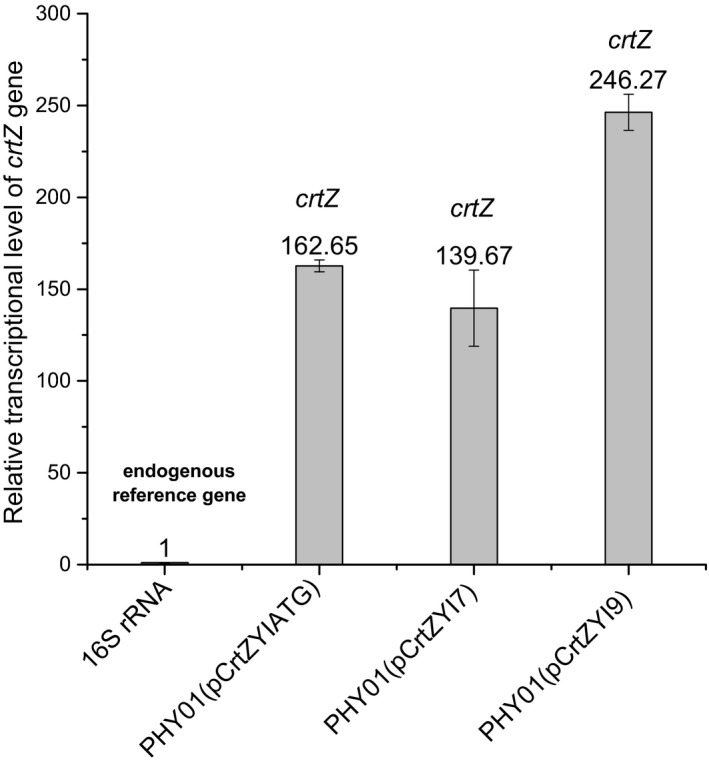
The relative transcriptional level of *crtZ* gene in the strains of control strain PHY01(pCrtZYIATG), and experimental strains of PHY01(pCrtZYI7) and PHY01(pCrtZYI9). 16S rRNA was used as the endogenous reference gene. The data were shown as mean ± standard deviation (*SD*) of three independent experiments

In order to investigate the relationship between non‐ATG initial codons and the transcriptional expression levels of the key carotenoid synthetic pathway genes, two representative strains PHY01(pCrtZYI7) and PHY01(pCrtZYI9) and the control strain PHY01(pCrtZYIATG) were chosen to analyze the strength of the gene expression through real‐time qPCR (RT‐qPCR). As indicated in the figures (Figures A5, A6, and A7), although with different initial codons, the transcription levels of the genes *crtI*, *crtY,* and *crtZ* were constant, which suggested that the non‐ATG codons did not affect the transcription levels of associated genes in *E. coli.*

**Figure A8:**
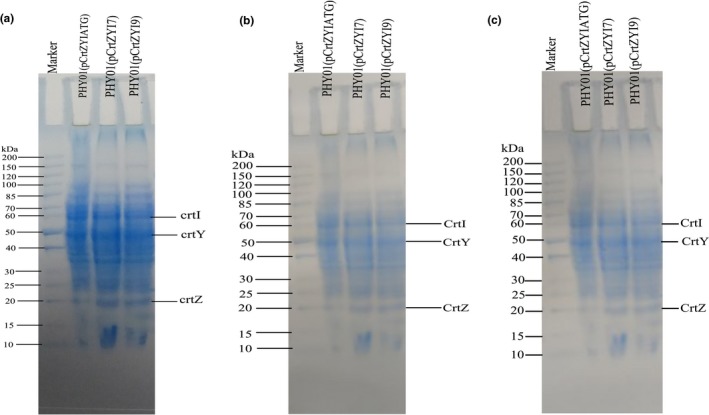
The SDS‐PAGE photograph of the proteins which were extracted from the control strain PHY01(pCrtZYIATG), and the experimental strains of PHY01(pCrtZYI7) and PHY01(pCrtZYI9). Figure (a–c) represented three repeated experiments

In the SDS‐PAGE experiment, as indicated in Figure A8 (a, b, and c), all three strains had their own corresponding bands matched the sizes of crtI, crtY, and crtZ proteins. Although it is difficult to distinguish clearly the brightness of crtI and crtY protein bands in the three strains, the crtZ protein band brightness of PHY01(pCrtZYI7) and PHY01(pCrtZYI9) was relative brighter than that of PHY01(pCrtZYIATG). These results indicated that in the higher zeaxanthin production, strains of PHY01(pCrtZYI7) and PHY01(pCrtZYI9) had higher crtZ protein expression than that of PHY01(pCrtZYIATG), suggested the non‐ATG initial codons affect the translation level expression of *crtZ* gene in *E. coli*.

The protein mass spectrometry was performed for determining whether the non‐ATG initial codons influence the gene translation levels expression in *E. coli*. The detailed protein mass spectrometry results of crtI, crtY, and crtZ were marked in red in Tables A7, A8, and A9. The emPAI value is used for relatively determine the protein quantity, whereas the values of Sum PEP Score, Score Sequest HT, and PSMs are used to detect the protein amount indirectly. It is demonstrated that the emPAI values of the crtZ protein in the strains of PHY01(pCrtZYI7) and PHY01(pCrtZYI9) were obviously higher than that of PHY01(pCrtZYIATG). The emPAI values of the crtY protein were nearly the same in the three strains, but the emPAI values of the crtI protein in the PHY01(pCrtZYIATG) were significantly higher than those of PHY01(pCrtZYI7) and PHY01(pCrtZYI9). Combined with RT‐qPCR data, these results proved that the non‐ATG initial codons indeed affected the gene expression in the translation level but not in the transcription levels in *E. coli*.

To understand how the different enzyme levels affect zeaxanthin production, protein mass spectrometry experiments were performed for control strain PHY01(pCrtZYIATG), which had original ATG initial codons for *crtZYI* genes, and two modulated hyper‐producing strains PHY01(pCrtZYI7) and PHY01(pCrtZYI9) with modulated initial codons. In the protein mass spectrometry results (Tables A7, A8, and A9), the quantity of detected proteins is represented by the emPAI value. It was determined from Tables A7, A8, and A9 that the emPAI values of crtZ from PHY01(pCrtZYI7) and PHY01(pCrtZYI9) exhibited 5.6‐ and 7.6‐fold increase relative to the control strain PHY01(pCrtZYIATG), respectively, while crtY emPAI values remain relatively steady for the three strains. And to our surprise, emPAI values of the first enzyme in the zeaxanthin pathway, crtI, dropped significantly compared with the control strain. Previous research reports demonstrated that the crtZ enzyme was the rate‐limit step and very essential for complete conversion from β‐carotene to zeaxanthin in the biosynthesis pathway of zeaxanthin (Nishizaki, Tsuge, Itaya, Doi, & Yanagawa, 2007; Pollmann, Breitenbach, & Sandmann, 2017). Thus, the fact that high‐production zeaxanthin strains PHY01(pCrtZYI7) and PHY01(pCrtZYI9) exhibited significant higher crtZ (β‐carotenoid hydroxylase) enzyme levels was consistent with the previous report (Ruther, Misawa, Böger, & Sandmann, 1997). However, the lower detected crtI enzyme levels in both zeaxanthin hyper‐producing strains PHY01(pCrtZYI7) and PHY01(pCrtZYI9) were not reported in related work, and we do not have a feasible explanation for it yet. However, this nonstraightforward case is worthy of investigation in future work. In addition, there is no report concerning modulating the expression of *crtZ*, *crtY,* and *crtI* simultaneously for regulating the production of zeaxanthin. Our findings here might give some clues for further optimizing the zeaxanthin synthetic pathway.

Although conventional promoter engineering is a common transcriptional regulation strategy, its disadvantages are as follows: (a) The promoters are long and have high sequence similarity, which might result in homologous recombination (Borodina & Nielsen, 2014); (b) when it is the inducible promoter, large amount of the expensive inducers were essential and inevitable for using these promoters; and (c) due to the promoter sequence is too long, and the promoter strategy is complicated and tedious to operate. As for RBS‐based engineering strategies, it still has some drawbacks: (a) Sometimes there are nonspecific interactions between the 30S subunit and mRNA (Seo, Kim, & Jung, 2012); and (b) the RBS sequence is relatively long and difficult to operate. Especially when combinatorial modulation techniques are performed, either promoter or RBS‐based strategies become more time‐consuming and complicated, due to several regulators are needed to be operated simultaneously.

Compared to the RBS or promoter engineering, the advantages of the CMIC approach are as follows: (a) For combinatorial modulation of several genes, the CMIC strategy costs the lowest experimental time and materials, due to only three nucleotides need to be operated for each gene, and it is feasible and has great flexibility; (b) operating the initial codons provides an extra layer for expression modulation in addition to promoters and RBSs, which might be used to further improve metabolic pathways already optimized by promoters and RBSs. And by our experiment, the improvement resulted from initial codon modulation was not marginal that the application of the CMIC strategy in *E. coli* resulting in nearly 10‐fold increased zeaxanthin production.

## 4 CONCLUSIONS

This study proves that changing only the three nucleotides of the initiation codons can be used to generate expression libraries with a large dynamic range and evenly distributed expression levels in *E. coli*. Based on these findings, the novel CMIC strategy was developed for metabolic pathway optimization and applied to the zeaxanthin synthesis pathway in *E. coli*. A combinatorial library was obtained containing strains with various zeaxanthin production levels, including a strain with a 10‐fold improvement over the starting strain. Therefore, CMIC was demonstrated to be a feasible technique for conveniently optimizing metabolic pathways. To our best knowledge, this is the first metabolic engineering strategy that manipulates the initiation codons for pathway optimization in *E. coli*.

The central principle and mechanism in all organisms have been researched to be highly conserved, and *E. coli* has been used as a model organism to have revealed many principles and mechanism in classic Genetics. Thus, we think the modulation with CMIC should be universally functional to some extent in other organisms. We plan to study this strategy in a model eukaryote, *Saccharomyces cerevisiae*, to determine whether such a modulation technique could be applied to eukaryotic systems and hope to present the work in the near future.

## CONFLICT OF INTERESTS

None declared.

## AUTHOR CONTRIBUTIONS

Changhao Bi conceptualized the study. Investigations, methodology, formal analysis, data curation, and project administration were carried out by Zaiqiang Wu. Supervision and validation were done by Junsong Wang, and funding acquisition and validation were provided by Xueli Zhang. Resources were provided by Dongdong Zhao and Siwei Li. Zaiqiang Wu wrote the original manuscript. Changhao Bi and Xueli Zhang reviewed and edited the manuscript. All authors approved the final version of this published article.

## ETHICS STATEMENT

None required.

## Data Availability

All data associated with the article have been included in this manuscript.

## APPENDIX 1

**Table A1:** *E. coli* strains and plasmids used in this study

Name	Characteristics	Sources
Strains		
DH5α	F‐Φ80lacZΔM15 Δ(lacZYA‐argF) U169 recA1 endA1 hsdR17(rk−, mk+) phoA supE44 thi‐1 gyrA96 relA1 λ‐	Invitrogen
MG1655	Wild type	Laboratory stored
M1‐46	ATCC8739, FRT‐Km‐FRT::M1‐46::lacZ	Lu et al. 2012
M1‐36	ATCC8739, FRT‐Km‐FRT::M1‐36::lacZ	Laboratory stored
PHY01	ATCC 8739, ldhA::RBSL9::crtEB, RBSL12::dxs, RBSL7::idi, M1‐46::sucAB, M1‐46::sdhABCD, M1‐46::talB, mRSL‐4:: ispG, mRSL‐14:: ispH	Laboratory stored
Plasmids		
pBBR1‐rfp	kan; pBBR1 replicon; RFP	Laboratory stored
p034apr	apr; pMB1 replicon	Laboratory stored
pQE60‐gfp	bla; pSC101 Origin1 replicon; GFP	Laboratory stored
pYL‐crtZYI	cat; pSC101 replicon; *crtZYI* genes cloned into pYL vector	Laboratory stored
pNNNrfp	kan, pBBR1 replicon; RFP‐NNN	This work
pNNNgfp	apr; pMB1 replicon; GFP‐NNN	This work
pNNNlacZ	apr; pMB1 replicon; lacZ‐NNN	This work
pCrtZYIATG	Cm; pSC101 replicon; crtZ‐ATG, crtY‐ATG, crtI‐ATG	This work
pCrtZYIlib	Cm, pSC101 replicon; crtZ‐NNN, crtY‐NNN, crtI‐NNN	This work

**Table A2:** Sequences of promoters BBa J23100, P46, and P36, and sequences of RBS of *rfp*, *gfp*, *lacZ,* and *crt ZYI*

Name	Sequences
BBa J23100	a **TTGACG**GCTAGCTCAGTCCTAGG**TACAGT**GCTAGC
RBS of *rfp*	TTTAAGAAGGAGATATACAT
P46	TTATCTCTGGCGGTG**TTGACA**AGAGATAACAACGTTGA**TATAAT**TGAGCCTCTCGCCCCACCAATTCGGTTTAAA
RBS of *gfp*	CCAGGAAACAGCT
RBS of *lacZ*	CCAGGAAACAGCT
P36	CAGAAAAAAAGATCAAAAAAATAC**TTGTGC**AAAAAATTGGGATCCC**TATAAT**GCGCCTCCGTTGAGACGAATAA
RBS of *crtZ*	CAATTTCACACAGGAGATCATAA
RBS of *crtY*	ATAAAGGAGGTAAAAT
RBS of *crtI*	ATAAAGGAGGATAATA

a The bold fonts represented −35 and −10 sequences in different promoters.

**Table A3:** Primers used in this study

Prime name	Sequence
Construction of pNNNrfp initiation codon library	
pBBR1‐rfp‐F	CCAGGTCTCAACATNNNGCGAGTAGCGAAGACG
pBBR1‐rfp‐R	CCAGGTCTCAATGTATATCTCCTTCTTAAAGCTAGCACTGTACC
Construction of pNNNgfp initiation codon library	
pMB1_apr_F	CCAGGTCTCAAGCGACAGATCGCTGAGATAG
pMB1_apr_R	CCAGGTCTCAGAGCAGACTTGACCTGATAG
P46‐up	CCAGGTCTCAGCTCTTATCTCTGGCGGTGTTGAC
GFP_RBS‐down	CCAGGTCTCATACCNNNAGCTGTTTCCTGGTT
Gfp_F	CCAGGTCTCAGGTAAGGGAGAAGAACTTTTCACTGG
Gfp_R	CCAGGTCTCACGCTCCGAGCGTTCTGAACAAATC
Construction of pNNNlacZ initiation codon library	
pMB1_apr_F	CCAGGTCTCA AGCG ACAGATCGCTGAGATAG
pMB1_apr_R	CCAGGTCTCA GAGC AGACTTGACCTGATAG
P46‐up	CCAGGTCTCA GCTC TTATCTCTGGCGGTGTTGAC
lacZ_RBS‐down	CCAGGTCTCA TGGT NNNAGCTGTTTCCTGGTT
LacZ_F	CCAGGTCTCA ACCA TGATTACGGATTCACTGGCCG
LacZ_R	CCAGGTCTCA CGCT ACGCGAAATACGGGCAGAC
Construction of pCrtZYIlib initiation codon library	
crt‐F	CCAGGTCTCACGCAGAAGCGGTCTGATAAAACAG
crt‐R	CCAGGTCTCAGTAGTGCCATTTACCCCCATTCAC
P36‐F	CCAGGTCTCACTAC CAGAAAAAAAGATCAAAAAAATAC
P36‐R	CCAGGTCTCAGATCTCCTGTGTGAAATTGTTATTCGT
crtZ‐F	CCAGGTCTCAGATCATAANNNTTGTGGATTTGGAATGCCCTG
crtZ‐R	CCAGGTCTCATACCTCCTTTATTTACTTCCCGGGTGGCGCGTCACG
crtY‐F	CCAGGTCTCAGGTAAAATNNNCCGCGGTATGATCTGATTCTGGTGG
crtY‐R	CCAGGTCTCAATCCTCCTTTATTTACATCGCCTGTTGACGGTGAGG
crtI‐F	CCAGGTCTCAGGATAATANNNAATAGAACTACAGTAATTGGCGCAGGCTT
crtI‐R	CCAGGTCTCATGCG TCAAGCCAGATCCTCCAGCATCAA

**Table A4:** Sequencing primers of plasmids and PCR products

Primer name	Sequence
Sequencing primers of pNNNrfp	
rfpp‐F	GAAGCCGGTCTTGTCGATCAGG
rfpp‐R	GGGCCGTTGAATCGGGATATGC
Sequencing primers of pNNNgfp and pNNNlacZ	
capr‐F	GGGCCGAGATCCGTTGAT
capr‐R	TCTTCACCTAGATCCTTT
Sequencing primers of pCrtZYIlib	
crtZ‐C	CTCGCAAGCTCGGGCAAA
crtY‐C	ATTCCGCTATGTTCCGCG
crtI‐C	CGCATGCTGAACCGTATG

**Table A5:** The primers that used for the real‐time qPCR

Primer name	Sequence
Primers of endogenous reference gene	
16S‐F	CATCCTGAACCACTGACCAG
16S‐R	AGCACCTTCACTTCCACG
Primers of crtI, crtY, and crtZ	
crtI‐qPCR‐F	GCCACTTCCTCAATCTATACCC
crtI‐qPCR‐R	TAATCCTGTTTTCCTGGGTCTC
crtY‐qPCR‐F	GGAGAGTGACGCAGTGATTG
crtY‐qPCR‐R	TGTAGACAAAGCGATAGCCTG
crtZ‐qPCR‐F	TGTTCTGGTCACTGTTATCGG
crtZ‐qPCR‐R	GTCATTAACCTCAAACCAGCC

**Table A6:** Initiation codons of *crtZ*, *crtY,* and *crtI* from selected strains from PHY01(pCrtZYIlib)

Strains	Initiation codon		
	*crtZ*	*crtY*	*crtI*
PHY01(pCrtZYIATG)	ATG	ATG	ATG
PHY01(pCrtZYI1)	TAG	AAC	ATG
PHY01(pCrtZYI2)	AAG	GAA	AAA
PHY01(pCrtZYI3)	CCA	CCT	ATG
PHY01(pCrtZYI4)	GGG	ACG	ATA
PHY01(pCrtZYI5)	AGC	ATG	GTA
PHY01(pCrtZYI6)	GTT	ATT	CTG
PHY01(pCrtZYI7)	TCA	GTG	TTG
PHY01(pCrtZYI8)	GAC	GTG	ACG
PHY01(pCrtZYI9)	ACG	ATT	CTG

**Table A7:** The protein mass spectrometry result for the determination of crtI, crtY, and crtZ protein expression by the engineered *E. coli* strain PHY01(pCrtZYIATG)

Accession	Description	Sum PEP Score	Coverage	#PSMs	#AAs	MW[kDa]	calc.PI	emPAI	Score Sequest HT
P0A698	UvrABC system protein A OS = *Escherichia coli* (strain K12)	42.24666618	7.97872	8	940	103.803	6.64	0.407	27.36333418
sp	006 CrtI	151.3272627	25.4065	19	492	54.769	6.77	3.52	102.4008672
P0A9C0	Anaerobic glycerol‐3‐phosphate dehydrogenase subunit A OS = Esc	68.23289629	27.1218	16	542	58.921	6.64	1.202	57.84568882
P0AB71	Fructose‐bisphosphate aldolase class 2 OS = *Escherichia coli*	33.73338241	24.5125	7	359	39.123	5.86	1.581	26.53024399
P11349	Respiratory nitrate reductase 1 beta chain OS = *Escherichia coli*	29.23611516	9.375	4	512	58.029	6.77	0.311	20.04963112
sp	007 CrtY	86.47902161	45.0777	16	386	43.604	8	3.962	63.83823073
P0A794	Pyridoxine 5′‐phosphate synthase OS = *Escherichia coli* (strain K12)	38.62064587	16.0494	5	243	26.368	5.95	1.276	25.20745921
P75691	Aldehyde reductase YahK OS = *Escherichia coli* (strain K12)	35.94943889	14.3266	6	349	37.954	6.23	0.995	24.27691317
P0AB77	2‐amino‐3‐ketobutyrate coenzyme A ligase OS = *Escherichia coli*	23.08778624	9.79899	4	398	43.09	5.97	0.551	15.84744815
sp	008 CrtZ	28.64109322	28.9763	8	175	20.168	9.9	1.287	58.87265334
P0AEC3	Aerobic respiration control sensor protein ArcB OS = *Escherichia coli*	4.008818242	1.41388	1	778	87.928	5.1	0.058	3.146479845
P77611	Electron transport complex subunit RsxC OS = *Escherichia*	7.615976868	7.2973	2	740	80.122	8.63	0.105	4.493131638

**Table A8:** The protein mass spectrometry result for the determination of crtI, crtY, and crtZ protein expression by the engineered *E. coli* strain PHY01(pCrtZYI7)

Accession	Description	Sum PEP Score	Coverage	#PSMs	#AAs	MW[kDa]	calc.PI	emPAI	Score Sequest HT
P0A698	UvrABC system protein A OS = *Escherichia coli* (strain K12)	42.24666618	7.97872	8	940	103.803	6.64	0.407	27.36333418
sp	006 CrtI	42.00374291	5.4878	5	492	54.769	6.77	0.487	26.96346498
P0A9C0	Anaerobic glycerol‐3‐phosphate dehydrogenase subunit A OS = Esc	108.0647275	32.6568	23	542	58.921	6.64	2.06	83.88820601
P0AB71	Fructose‐bisphosphate aldolase class 2 OS = *Escherichia coli*	97.09414663	29.2479	11	359	39.123	5.86	3.437	53.46555638
P11349	Respiratory nitrate reductase 1 beta chain OS = *Escherichia coli*	86.5361797	24.6094	16	512	58.029	6.77	1.581	63.89301682
sp	007 CrtY	72.35807128	40.1237	12	386	43.604	8	3.276	51.25720981
P0A794	Pyridoxine 5′‐phosphate synthase OS = *Escherichia coli* (strain K12)	65.53282216	16.4609	7	243	26.368	5.95	2.162	39.8662715
P75691	Aldehyde reductase YahK OS = *Escherichia coli* (strain K12)	57.03702369	23.7822	10	349	37.954	6.23	1.512	45.17795205
P0AB77	2‐amino‐3‐ketobutyrate coenzyme A ligase OS = *Escherichia coli*	46.90887079	20.603	8	398	43.09	5.97	1.404	35.01567221
sp	008 CrtZ	198.3765561	41.5692	8	175	20.168	9.9	7.256	227.63115
P0AEC3	Aerobic respiration control sensor protein ArcB OS = *Escherichia coli*	4.97510404	1.41388	1	778	87.928	5.1	0.058	3.045333862
P77611	Electron transport complex subunit RsxC OS = *Escherichia*	3.865271223	4.86486	2	740	80.122	8.63	0.105	3.155004501

**Table A9:** The protein mass spectrometry result for the determination of crtI, crtY, and crtZ protein expression by the engineered *E. coli* strain PHY01(pCrtZYI9)

Accession	Description	Sum PEP Score	Coverage	#PSMs	#AAs	MW[kDa]	calc.PI	emPAI	Score sequest HT
P0A698	UvrABC system protein A OS = *Escherichia coli* (strain K12)	47.00549802	9.57447	10	940	103.803	6.64	0.407	35.69448233
sp	006 CrtI	37.11252812	7.98889	7	492	54.769	6.77	0.398	23.78128947
P0A9C0	Anaerobic glycerol‐3‐phosphate dehydrogenase subunit A OS = Esc	61.79982658	23.2472	12	542	58.921	6.64	0.931	45.54526341
P0AB71	Fructose‐bisphosphate aldolase class 2 OS = *Escherichia coli*	32.91694772	29.2479	7	359	39.123	5.86	1.581	25.66632211
P11349	Respiratory nitrate reductase 1 beta chain OS = *Escherichia coli*	41.07101889	17.3828	6	512	58.029	6.77	0.501	27.12277055
sp	007 CrtY	48.49429593	12.9534	10	386	43.604	8	2.15	33.47542071
P0A794	Pyridoxine 5’‐phosphate synthase OS = *Escherichia coli* (strain K12)	57.17310024	29.2181	9	243	26.368	5.95	3.394	40.71503448
P75691	Aldehyde reductase YahK OS = *Escherichia coli* (strain K12)	51.91666791	23.4957	8	349	37.954	6.23	1.512	34.80960083
P0AB77	2‐amino‐3‐ketobutyrate coenzyme A ligase OS = *Escherichia coli*	5.276033776	10.5528	3	398	43.09	5.97	0.389	4.364562869
sp	008 CrtZ	297.3973216	36.7087	10	175	20.168	9.9	9.849	356.2761002
P0AEC3	Aerobic respiration control sensor protein ArcB OS = *Escherichia coli*	15.77476879	6.94087	4	778	87.928	5.1	0.252	12.87201095
P77611	Electron transport complex subunit RsxC OS = *Escherichia*	12.40281299	4.86486	2	740	80.122	8.63	0.105	7.91286993

## References

[mbo3930-bib-0001] Aiba, H. , Mori, K. , Tanaka, M. , Ooi, T. , Roy, A. , & Danchin, A. (1984). The complete nucleotide sequence of the adenylate cyclase gene of *Escherichia coli* . Nucleic Acids Research, 12(24), 9427–9440. 10.1093/nar/12.24.9427 6393056 PMC320471

[mbo3930-bib-0002] Avalos, J. L. , Fink, G. R. , & Stephanopoulos, G. (2013). Compartmentalization of metabolic pathways in yeast mitochondria improves the production of branched‐chain alcohols. Nature Biotechnology, 31(4), 335–341. 10.1038/nbt.2509 PMC3659820 23417095

[mbo3930-bib-0003] Barbirato, F. , Grivet, J. P. , Soucaille, P. , & Bories, A. (1996). 3‐hydroxypropionaldehyde, an inhibitory metabolite of glycerol fermentation to 1, 3‐propanediol by enterobacterial species. Applied and Environmental Microbiology, 62(4), 1448–1451. 10.1016/0921-8777(95)00057-7 8919810 PMC167915

[mbo3930-bib-0004] Beard, C. W. , & Spindler, K. R. (1996). Analysis of early region 3 mutants of mouse adenovirus type 1. Journal of Virology, 70(9), 5867–5874. 10.1016/S0360-3016(02)03031-6 8709206 PMC190604

[mbo3930-bib-0005] Berry, A. , Dodge, T. C. , Pepsin, M. , & Weyler, W. (2002). Application of metabolic engineering to improve both the production and use of biotech indigo. Journal of Industrial Microbiology and Biotechnology, 28(3), 127–133. 10.1038/sj/jim/7000228 12074085

[mbo3930-bib-0006] Borodina, I. , & Nielsen, J. (2014). Advances in metabolic engineering of yeast *Saccharomyces cerevisiae* for production of chemicals. Biotechnology Journal, 9(5), 609–620. 10.1002/biot.201300445 24677744

[mbo3930-bib-0007] Bourcier de Carbon, C. , Thurotte, A. , Wilson, A. , Perreau, F. , & Kirilovsky, D. (2015). Biosynthesis of soluble carotenoid holoproteins in *Escherichia coli* . Scientific Reports, 5, 9085 10.1038/srep09085 25765842 PMC4358027

[mbo3930-bib-0008] Brynildsen, M. P. , Wong, W. W. , & Liao, J. C. (2005). Transcriptional regulation and metabolism. Biochemical Society Transactions, 33(6), 1423–1426. 10.1042/BST20051423 16246136

[mbo3930-bib-0009] Chen, H. , Bjerknes, M. , Kumar, R. , & Jay, E. (1994). Determination of the optimal aligned spacing between the Shine–Dalgarno sequence and the translation initiation codon of *Escherichia coli* mRNAs. Nucleic Acids Research, 22(23), 4953–4957. 10.1093/nar/22.23.4953 7528374 PMC523762

[mbo3930-bib-0010] Chen, M. , Zhang, L. , Zhang, H.‐Y. , Xiong, X. , Wang, B. , Du, Q. , … Liang, Z. (2005). A universal plasmid library encoding all permutations of small interfering RNA. Proceedings of the National Academy of Sciences of the United States of America, 102(7), 2356–2361. 10.1073/pnas.0401549101 15695593 PMC548965

[mbo3930-bib-0011] Cox, R. S. 3rd , Surette, M. G. , & Elowitz, M. B. (2007). Programming gene expression with combinatorial promoters. Molecular Systems Biology, 3, 145 10.1038/msb4100187 18004278 PMC2132448

[mbo3930-bib-0012] Danchin, A. , Guiso, N. , Roy, A. , & Ullmann, A. (1984). Identification of the *Escherichia coli cya* gene product as authentic adenylate cyclase. Journal of Molecular Biology, 175(3), 403–408. 10.1016/0022-2836(84)90356-5 6427472

[mbo3930-bib-0013] Espadas, G. , Borras, E. , Chiva, C. , & Sabido, E. (2017). Evaluation of different peptide fragmentation types and mass analyzers in data‐dependent methods using an Orbitrap Fusion Lumos Tribrid mass spectrometer. Proteomics, 17(9), 10.1002/pmic.201600416 28266123

[mbo3930-bib-0014] Farhi, M. , Marhevka, E. , Masci, T. , Marcos, E. , Eyal, Y. , Ovadis, M. , … Vainstein, A. (2011). Harnessing yeast subcellular compartments for the production of plant terpenoids. Metabolic Engineering, 13(5), 474–481. 10.1016/j.ymben.2011.05.001 21601648

[mbo3930-bib-0015] Farmer, W. R. , & Liao, J. C. (2000). Improving lycopene production in *Escherichia coli* by engineering metabolic control. Natural Biotechnology, 18(5), 533–537. 10.1038/75398 10802621

[mbo3930-bib-0016] Harcum, S. W. , & Bentley, W. E. (1999). Heat‐shock and stringent responses have overlapping protease activity in *Escherichia coli* . Applied Biochemistry and Biotechnology, 80(1), 23–37. 10.1385/ABAB:80:1:23 10394618

[mbo3930-bib-0017] Hecht, A. , Glasgow, J. , Jaschke, P. R. , Bawazer, L. A. , Munson, M. S. , Cochran, J. R. , … Salit, M. E. , (2017). Measurements of translation initiation from all 64 codons in *E. coli* . Nucleic Acids Research, 45(7), 3615–3626. 10.1093/nar/gkx070 28334756 PMC5397182

[mbo3930-bib-0018] Hillson, N. J. , Rosengarten, R. D. , & Keasling, J. D. (2012). J5 DNA assembly design automation software. ACS Synthetic Biology, 1(1), 14–21. 10.1021/sb2000116 23651006

[mbo3930-bib-0019] Jin, E. , Wong, L. , Jiao, Y. , Engel, J. , Holdridge, B. , & Xu, P. (2017). Rapid evolution of regulatory element libraries for tunable transcriptional and translational control of gene expression. Synthetic and System Biotechnology, 2(4), 295–301. 10.1016/j.synbio.2017.10.003 PMC5851936 29552654

[mbo3930-bib-0020] Juminaga, D. , Baidoo, E. E. K. , Redding‐Johanson, A. M. , Batth, T. S. , Burd, H. , Mukhopadhyay, A. , … Keasling, J. D. (2012). Modular engineering of L‐tyrosine production in *Escherichia coli* . Applied Environmental Microbiology, 78(1), 89–98. 10.1128/AEM.06017-11 22020510 PMC3255607

[mbo3930-bib-0021] Keasling, J. D. (2010). Manufacturing molecules through metabolic engineering. Science, 330(6009), 1355–1358. 10.1126/science.1193990 21127247

[mbo3930-bib-0022] Krinsky, N. I. , & Johnson, E. J. (2005). Carotenoid actions and their relation to health and disease. Molecular Aspects of Medicine, 26(6), 459–516. 10.1016/j.mam.2005.10.001 16309738

[mbo3930-bib-0023] Kumari, S. , Panesar, P. S. , & Panesar, R. (2004). Production of ß‐galactosidase using novel yeast isolate from whey. International Journal of Dairy Science, 6(2), 150–157.

[mbo3930-bib-0024] Leonard, E. , Lim, K. H. , Saw, P. N. , & Koffas, M. A. (2007). Engineering central metabolic pathways for high‐level flavonoid production in *Escherichia coli* . Applied and Environmental Microbiology, 73(12), 3877–3886. 10.1128/AEM.00200-07 17468269 PMC1932724

[mbo3930-bib-0025] Li, Q. , Fan, F. , Gao, X. , Yang, C. , Bi, C. , Tang, J. , … Zhang, X. (2017). Balanced activation of IspG and IspH to eliminate MEP intermediate accumulation and improve isoprenoids production in *Escherichia coli* . Metabolic Engineering, 44, 13–21. 10.1016/j.ymben.2017.08.005 28864262

[mbo3930-bib-0026] Li, S. , Zhou, Y. , Xiao, K. , Li, J. , & Tian, Z. (2018). Selective fragmentation of the N‐glycan moiety and protein backbone of ribonuclease B on an Orbitrap Fusion Lumos Tribrid Mass Spectrometer. Rapid Communications in Mass Spectrometry, 32(23), 2031–2039. 10.1002/rcm.8273 30152909

[mbo3930-bib-0027] Looman, A. C. , Bodlaender, J. , Comstock, L. J. , Eaton, D. , Jhurani, P. , Boer, H. A. , & van Knippenberg, P. H. (1987). Influence of the codon following the AUG initiation codon on the expression of a modified *lacZ* gene in *Escherichia coli* . The EMBO Journal, 6(8), 2489–2492. 10.1002/j.1460-2075.1987.tb02530.x 3311730 PMC553658

[mbo3930-bib-0028] Lu, J. , Tang, J. , Liu, Y. I. , Zhu, X. , Zhang, T. , & Zhang, X. (2012). Combinatorial modulation of *galp* and *glk* gene expression for improved alternative glucose utilization. Applied Microbiology and Biotechnology, 93, 2455–2462. 10.1007/s00253-011-3752-y 22159736

[mbo3930-bib-0029] Moeller, S. M. , Jacques, P. F. , & Blumberg, J. B. (2000). The potential role of dietary *Xanthophylls* in cataract and age‐related macular degeneration. Journal of the American College of Nutrition, 19(Suppl 5), 522S–527S. 10.1080/07315724.2000.10718975 11023002

[mbo3930-bib-0030] Nishino, H. , Murakoshi, M. , Tokuda, H. , & Satomi, Y. (2009). Cancer prevention by carotenoids. Archives of Biochemistry and Biophysics, 483(2), 165–168. 10.1016/j.abb.2008.09.011 18848517

[mbo3930-bib-0031] Nishizaki, T. , Tsuge, K. , Itaya, M. , Doi, N. , & Yanagawa, H. (2007). Metabolic engineering of carotenoid biosynthesis in *Escherichia coli* by ordered gene assembly in *Bacillus subtilis* . Applied Environmental Microbiology, 73(4), 1355–1361. 10.1128/AEM.02268-06 17194842 PMC1828653

[mbo3930-bib-0032] O'Connor, M. , Gregory, S. , Rajbhandary, U. T. , & Dahlberg, A. (2001). Altered discrimination of start codons and initiator tRNAs by mutant initiation factor 3. RNA, 7(7), 969–978. 10.1017/S1355838201010184 11453069 PMC1370149

[mbo3930-bib-0033] Otsuka, M. , Nakabeppu, U. , & Sekiguchi, M. (1985). Ability of various alkylating agents to induce adaptive and SOS responses: A study with lacZ fusion. Mutation Research DNA Repair Reports, 146(2), 149–154. 10.1016/0167-8817(85)90005-7 2993877

[mbo3930-bib-0034] Pfleger, B. F. , Pitera, D. J. , Smolke, C. D. , & Keasling, J. D. (2006). Combinatorial engineering of intergenic regions in operons tunes expression of multiple genes. Nature Biotechnology, 24(8), 1027–1032. 10.1038/nbt1226 16845378

[mbo3930-bib-0035] Pitera, D. J. , Paddon, C. J. , Newman, J. D. , & Keasling, J. D. (2007). Balancing a heterologous mevalonate pathway for improved isoprenoid production in *Escherichia coli* . Metabolic Engineering, 9(2), 193–207. 10.1016/j.ymben.2006.11.002 17239639

[mbo3930-bib-0036] Pollmann, H. , Breitenbach, J. , & Sandmann, G. (2017). Engineering of the carotenoid pathway in *Xanthophyllomyces dendrorhous* leading to the synthesis of zeaxanthin. Applied Microbiology and Biotechnology, 101(1), 103–111. 10.1007/s00253-016-7769-0 27527661

[mbo3930-bib-0037] Reddy, P. , Peterkofsky, A. , & McKenney, K. (1985). Translational efficiency of the *Escherichia coli* adenylate cyclase gene: Mutating the UUG initiation codon to GUG or AUG results in increased gene expression. Proceedings of the National Academy of Sciences of the United States of America, 82(17), 5656–5660. 10.1073/pnas.82.17.5656 3898067 PMC390610

[mbo3930-bib-0038] Rhee, S. , Yang, S. J. , Lee, S. J. , & Park, D. (2004). BetaPix‐b(L), a novel isoform of betaPix, is generated by alternative translation. Biochemical and Biophysical Research Communication, 318(2), 415–421. 10.1016/j.bbrc.2004.04.039 15120616

[mbo3930-bib-0039] Ruther, A. , Misawa, N. , Böger, P. , & Sandmann, G. (1997). Production of zeaxanthin in *Escherichia coli* transformed with different carotenogenic plasmids. Applied Microbiology & Biotechnology, 48(2), 162–167. 10.1007/s002530051032 9299773

[mbo3930-bib-0040] Sajilata, M. G. , Singhal, R. S. , & Kamat, M. Y. (2008). The carotenoid pigment zeaxanthin – A review. Comprehensive Reviews in Food Science and Food Safety, 7(1), 29–49. 10.1111/j.1541-4337.2007.00028.x

[mbo3930-bib-0041] Salis, H. M. , Mirsky, E. A. , & Voigt, C. A. (2009). Automated design of synthetic ribosome binding sites to control protein expression. Nature Biotechnology, 27(10), 946–950. 10.1038/nbt.1568 PMC2782888 19801975

[mbo3930-bib-0042] Seo, S. W. , Kim, S. C. , & Jung, G. Y. (2012). Synthetic regulatory tools for microbial engineering. Biotechnology and Bioprocess Engineering, 17(1), 1–7. 10.1007/s12257-011-0563-z

[mbo3930-bib-0043] Sies, H. , & Stahl, W. (1998). Lycopene: Antioxidant and biological effects and its bioavailability in the human. Proceedings of the Society for Experimental Biology and Medicine, 218(2), 121–124. 10.3181/00379727-218-44285a 9605209

[mbo3930-bib-0044] Stahl, W. , & Sies, H. (2005). Bioactivity and protective effects of natural carotenoids. Biochimica Et Biophysica Acta (BBA) ‐ Molecular Basis of Disease, 1740(2), 101–107. 10.1016/j.bbadis.2004.12.006 15949675

[mbo3930-bib-0045] Stenström, C. M. , Holmgren, E. , & Isaksson, L. A. (2001). Cooperative effects by the initiation codon and its flanking regions on translation initiation. Gene, 273(2), 259–265. 10.1016/S0378-1119(01)00584-4 11595172

[mbo3930-bib-0046] Sun, T. , Miao, L. , Li, Q. , Dai, G. , Lu, F. , Liu, T. , … Ma, Y. (2014). Production of lycopene by metabolically‐engineered *Escherichia coli* . Biotechnology Letters, 36(7), 1515–1522. 10.1007/s10529-014-1543-0 24806808

[mbo3930-bib-0047] Sussman, J. K. , Simons, E. L. , & Simons, R. W. (1996). *Escherichia coli* translation initiation factor 3 discriminates the initiation codon in vivo. Molecular Microbiology, 21(2), 347–360. 10.1046/j.1365-2958.1996.6371354.x 8858589

[mbo3930-bib-0048] Tang, L. , Morris, J. , Wan, J. , Moore, C. , Fujita, Y. , Gillaspie, S. , … Asano, K. (2017). Competition between translation initiation factor eIF5 and its mimic protein 5MP determines non‐ATG initiation rate genome‐wide. Nucleic Acids Research, 45(20), 11941–11953. 10.1093/nar/gkx808 28981728 PMC5714202

[mbo3930-bib-0049] Thomson, L. R. , Toyoda, Y. , Langner, A. , Delori, F. C. , Garnett, K. M. , Craft, N. , … Dorey, C. K. (2002). Elevated retinal zeaxanthin and prevention of light‐induced photoreceptor cell death in quail. Investigative Ophthalmol and Visual Science, 43(11), 3538–3549. 10.1007/s00417-002-0565-9 12407166

[mbo3930-bib-0050] Watts, K. T. , Mijts, B. N. , & Schmidt‐Dannert, C. (2005). Current and emerging approaches for natural product biosynthesis in microbial cells. Advanced Synthesis & Catalysis, 347(7–8), 927–940. 10.1002/adsc.200505062

[mbo3930-bib-0051] Whitehead, A. J. , Mares, J. A. , & Danchin, R. P. (2006). Macular pigment: A review of current knowledge. Archives of Ophthalmology, 124(7), 1038–1045. 10.1001/archopht.124.7.1038 16832030

[mbo3930-bib-0052] Wu, G. , Yan, Q. , Jones, J. A. , Tang, Y. J. , Fong, S. S. , & Koffas, M. A. G. (2016). Metabolic burden: Cornerstones in synthetic biology and metabolic engineering applications. Trends in Biotechnology, 34(8), 652–664. 10.1016/j.tibtech.2016.02.010 26996613

[mbo3930-bib-0053] Xu, P. , Bhan, N. , & Koffas, M. A. (2013). Engineering plant metabolism into microbes: From systems biology to synthetic biology. Current Opinion Biotechnology, 24(2), 291–299. 10.1016/j.copbio.2012.08.010 22985679

[mbo3930-bib-0054] Xu, P. , Gu, Q. , Wang, W. , Wong, L. , Bower, A. G. , Collins, C. H. , & Koffas, M. A. (2013). Modular optimization of multi‐gene pathways for fatty acids production in *E. coli* . Nature Communications, 4, 1409 10.1038/ncomms2425 23361000

[mbo3930-bib-0055] Xu, P. , Li, L. , Zhang, F. , Stephanopoulos, G. , & Koffas, M. (2014). Improving fatty acids production by engineering dynamic pathway regulation and metabolic control. Proceedings of the National Academy of Sciences of the United States of America, 111(31), 11299–11304. 10.1073/pnas.1406401111 25049420 PMC4128127

[mbo3930-bib-0056] Xu, P. , Rizzoni, E. A. , Sul, S. Y. , & Stephanopoulos, G. (2017). Improving metabolic pathway efficiency by statistical model‐based multivariate regulatory metabolic engineering. ACS Synthetic Biology, 6(1), 148–158. 10.1021/acssynbio.6b00187 27490704

[mbo3930-bib-0057] Xu, P. , Vansiri, A. , Bhan, N. , & Koffas, M. A. (2012). Epathbrick: A synthetic biology platform for engineering metabolic pathways in *E. coli* . ACS Synthetic Biology, 1(7), 256–266. 10.1021/sb300016b 23651248

[mbo3930-bib-0058] Zaslaver, A. , Bren, A. , Ronen, M. , Itzkovitz, S. , Kikoin, I. , Shavit, S. , … Alon, U. (2006). A comprehensive library of fluorescent transcriptional reporters for *Escherichia coli* . Nature Methods, 3(8), 623–628. 10.1038/nmeth895 16862137

[mbo3930-bib-0059] Zhang, F. , Carothers, J. M. , & Keasling, J. D. (2012). Design of a dynamic sensor‐regulator system for production of chemicals and fuels derived from fatty acids. Nature Biotechnology, 30(4), 354–359. 10.1038/nbt.2149 22446695

[mbo3930-bib-0060] Zhao, J. , Li, Q. , Sun, T. , Zhu, X. , Xu, H. , Tang, J. , … Ma, Y. (2013). Engineering central metabolic modules of *Escherichia coli* for improving beta‐carotene production. Metabolic Engineering, 17, 42–50. 10.1016/j.ymben.2013.02.002 23500001

[mbo3930-bib-0061] Zhu, M. M. , Lawman, P. D. , & Cameron, D. C. (2002). Improving 1, 3‐propanediol production from glycerol in a metabolically engineered *Escherichia coli* by reducing accumulation of sn‐glycerol‐3‐phosphate. Biotechnology Progress, 18(4), 694–699. 10.1021/bp020281 12153300

[mbo3930-bib-0062] Zhu, X. , Zhao, D. , Qiu, H. , Fan, F. , Man, S. , Bi, C. , & Zhang, X. (2017). CRISPR/Cas9‐facilitated multiplex pathway optimization (CFPO) technique and its application to improve the *Escherichia coli* xylose utilization pathway. Metabolic Engineering, 43, 37–45. 10.1016/j.ymben.2017.08.003 28800965

